# A systematic review of studies measuring health-related quality of life of general injury populations: update 2010–2018

**DOI:** 10.1186/s12955-020-01412-1

**Published:** 2020-05-29

**Authors:** A. J. L. M. Geraerds, Amy Richardson, Juanita Haagsma, Sarah Derrett, Suzanne Polinder

**Affiliations:** 1 Department of Public Health, Erasmus MC, University Medical Center Rotterdam, P.O. Box 2040, 3000 Rotterdam, CA The Netherlands; 2 Injury Prevention Research Unit, Department of Preventive and Social Medicine, Dunedin School of Medicine, University of Otago, Dunedin, New Zealand

**Keywords:** Health-related quality of life, Injuries, Systematic review

## Abstract

**Background:**

Studies examining the impact of injury on health-related quality of life (HRQL) over time are necessary to understand the short- and long-term consequences of injury for population health. The aim of this systematic review was to provide an evidence update on studies that have measured HRQL over time in general injury populations using a generic (general) health state measure.

**Methods:**

Studies conducted between 2010 and 2018 that assessed HRQL at more than one time point among general injury populations were eligible for inclusion. Two reviewers independently extracted information from each study on design, HRQL measure used, method of HRQL measure administration, timing of assessment(s), predictive variables, ability to detect change, and findings. Quality appraisals of each study were also completed by two reviewers using items from the RTI Item Bank on Risk of Bias and Precision of Observational Studies and the Guidelines for the Conduction of Follow-up Studies Measuring Injury-Related Disability.

**Results:**

Twenty-nine studies (44 articles) that met the inclusion criteria were identified. HRQL was measured using 14 different generic measures; the SF-36, SF-12, and EQ-5D were used most frequently. A varying number of follow-up assessments were undertaken, ranging from one to five. Follow-up often occurred 12 months post-injury. Fewer studies (*n* = 11) examined outcomes two or more years post-injury, and only one to 10 years post-injury. While most studies documented improvements in HRQL over time since the injury event, study populations had not returned to pre-injury status or reached general population norm HRQL values at post-injury follow-ups.

**Conclusions:**

Since 2010 there has been a substantial increase in the number of studies evaluating the HRQL of general injury populations. However, significant variability in study design continues to impede quantification of the impact of injury on population health over time. Variation between studies is particularly evident with respect to timing and number of follow-up assessments, and selection of instruments to evaluate HRQL.

## Background

Worldwide, the global burden of disability continues to increase as a consequence of population growth, reductions in mortality due to improvements in healthcare, and the ageing of populations [1]. This presents a significant challenge for health systems which face growing demand for services designed to reduce the impact of disability on quality of life [2]. Injury has been identified as a key contributor to the global disability burden, particularly in high and middle-income countries [1]. Despite a notable decline in deaths from injury over time, non-fatal injuries remain a leading cause of hospitalisation [3]. The age-adjusted annualised rate of injuries requiring some form of medical treatment was approximately 126 per 1000 members of the United States (US) population in 2014 [4]. Current information regarding the impact of injury on subsequent disability is essential to plan for the effective allocation of available resources within health systems in order to promote optimum recovery from injury. This information can also be disseminated to patients to ensure they have accurate expectations for their recovery, and may be useful in the development of targeted interventions designed to minimise disability after injury.

While some information is available on the incidence of both fatal and nonfatal injuries, these data do not adequately depict the long-term consequences for injured individuals [3]. As a result, measures of health-related quality of life (HRQL), often assessing functional status (an important component of disability) [5] are increasingly utilised to quantify the effect of injury on population health [6]. HRQL measures, including generic and disease-specific measures, aim to provide a comprehensive estimation of health, and are often self-reported [7]. When examining outcomes following injury it is useful to use generic HRQL measures as these enable comparison of outcomes and recovery patterns within and between different injury populations [8]. Such measures also allow for comparisons between injured individuals and members of the general population, and with people with other health conditions [9]. This information can be used to inform approaches to rehabilitation and effective community reintegration.

Most generic HRQL measures are comprised of items that aim to measure health in relation to a broad range of dimensions, such as physical health, psychological health, mobility, social relationships, and environmental health [10]. There are different approaches to the reporting of findings obtained using these measures. Some studies report the proportion of individuals experiencing difficulties with respect to particular HRQL dimensions, while others report summary scores for each dimension (e.g. means and standard deviations/confidence intervals), and/or a global HRQL score based on the sum of all items within the measure. Some measures derive utility scores (weights) which are often determined by asking members of the general population to provide their ‘preferences’ for certain health states. Utility scores are commonly used in economic evaluations, incorporating the impact of injury on both quantity and quality of life [11]. Although there are various approaches to reporting findings from measures of HRQL, each approach can be used to understand patterns of HRQL over time for people with a broad range of injuries, highlighting potential pathways to recovery.

An earlier systematic review was conducted to examine studies that had measured HRQL using a generic instrument among general injury populations, in order to summarise existing knowledge in this area [12]. The review included studies conducted during 1995–2009 and found a lack of consensus on preferred HRQL instruments and study designs for the measurement of injury-related outcomes [12]. A total of 24 different generic HRQL and functional status measures were identified in the 41 studies meeting inclusion criteria. The most frequently used measures included the Medical Outcome Study Short Form-36 items (SF-36), the Functional Independence Measure (FIM), the Glasgow Outcome Scale (GOS), and the EQ-5D-3 L. These measures were found to be administered at a range of different times points post-injury, with follow-up most commonly occurring at 6, 12 and 24 months. Twelve studies reported HRQL utility scores. Overall, studies found that while significant recovery occurred in the first year post-injury, deficits from full recovery continued up to 2 years post-injury (when compared with population norms or pre-injury health status) [12]. This was observed among populations with a broad range of injury severities, as well as severely injured populations.

Given the increasingly recognised importance of documenting the HRQL outcomes experienced by specific subpopulations, including individuals with injury [13], it is expected that many additional studies will have used generic health state measures among general injury populations since 2009 [14, 15]. However, it is unclear exactly how many studies have been conducted, how studies reported HRQL findings, and whether there has been greater consistency in study designs (including use of HRQL instruments, study populations, and assessment time points). It is possible that greater consistency in study designs may have been facilitated by the publication of the European Consumer Safety Association guidelines for undertaking follow-up studies measuring injury-related disability in 2007 [16]. These guidelines recommend the use of both the EQ-5D and Health Utilities Mark III (HUI) in all studies examining injury-related disability, with assessments at 1, 2, 4 and 12 months post-injury in addition to a pre-injury assessment. The earlier systematic review concluded that the guidelines were not being followed; yet this may have been because included studies had already finalised their protocol and/or data collection prior to the publication of the guidelines.

In order to gain contemporary information on injury outcomes and to investigate whether there has been an increase in the consistency of study designs since 2009 we conducted an updated systematic review of studies measuring HRQL with a generic instrument in general injury populations. Increased consistency in study designs would allow for improved comparisons between studies and increased precision in estimates of the burden of injury over time. As in the earlier review, we aimed to identify: i) which generic HRQL measures were used; ii) what methods were used to administer the measures; iii) the time points at which HRQL was measured; iv) how HRQL findings were reported; and v) whether changes over time, and predictors of, HRQL were assessed. We also explored whether studies eligible for inclusion used HRQL measures with properties that meet widely accepted recommendations in the field (with respect to internal consistency, reliability, measurement error, content validity, construct validity, criterion validity, responsiveness, and interpretability) [17]. Studies using appropriate measures and consistent designs are essential to ensure that accurate information on the burden of injury is available, allowing for the effective targeting of resources to maintain HRQL after injury.

## Methods

### Data sources and strategy

A new search of empirical studies on the HRQL of general injury populations was conducted. The search strategy that was developed for the systematic review of Polinder et al. [12] was updated in collaboration with a librarian specialising in literature searches. In order to match the database specific indexing terms, the search strategy was adjusted for the different electronic databases: Embase, PubMed (Medline Ovid), Web of Science and PsycINFO. The terms used in the search strategy were: ‘quality of life’ and ‘health related quality of life’, ‘functional status assessment’, ‘injury’ and ‘trauma’, and ‘cohort analysis’ (complete search strategy in Appendix 1). Articles were included in the search if the period of publication was between 2010 and 2018, and if they were peer-reviewed. The reference lists of the included articles were also screened, in order to detect additional articles that were relevant, and to identify important key terms. Details of the systematic review process were successfully registered and published within the PROSPERO database (registration number CRD42019120207).

### Selection criteria

To be included in this review, studies had to use a generic HRQL or disability measure at more than one time point in a population of injury/trauma patients. While HRQL and disability are unique constructs, the World Health Organization International Classification of Functioning, Disability and Health (ICF) acknowledges the relationship between disability and HRQL, particularly with respect to participation in activities of daily living [5]. For the purpose of this review, the World Health Organization (WHO) definition of disability is used. The WHO defines disability as an umbrella term reflecting impairments, activity limitations, and participation restrictions [18]. The concept of HRQL is more specific, reflecting an individual’s or population’s perceptions of health (mental and physical) and functional status [19]. Several measures of disability, such as the World Health Organization Disability Assessment Schedule (WHODAS) based on the ICF, can be used to evaluate not only disability but also HRQL [20].

Additional inclusion criteria were publication in English and in a peer-reviewed journal between 2010 and 2018. Studies that focused on only one specific injury population, such as traumatic brain injury patients, were excluded as only studies with a general injury population were the focus of this review. Furthermore, studies measuring HRQL in people other than individuals with injury were excluded, as were studies employing non-generic HRQL instruments, and review and pilot studies. There was no restriction on age or injury severity. Therefore, studies focusing on a specific age group or specific injury severity, but not focusing on a specific injury, were included.

### Data extraction and quality assessment

After completion of the database searches, relevant articles were selected in three steps. First, the titles of the articles were screened, next, the abstracts of the articles selected in step one were screened, and finally, the entire articles selected in step two were read. By screening the titles, abstracts and articles, it was determined whether an article should be included or not according to the selection criteria. The screening procedure was conducted by two researchers independently (AG and AR). In cases of disagreement between the two researchers, a third researcher (JH) was consulted. This researcher also checked a sample of abstracts (*n* = 50) in order to quality assure the process. The full articles that were eligible for inclusion were then analysed by two reviewers (AG and AR), using a modified version of the data extraction form developed for the original review by Polinder et al. [12]

The methodological quality of each study was independently assessed by two researchers (AG and AR) using three items from the RTI Item Bank on Risk of Bias and Precision of Observational Studies [21]. This item bank consists of 29 items designed to evaluate the quality of observational studies of interventions or exposures. It is recommended to select items that can evaluate the most critical threats to validity associated with the studies under investigation. For this review, items 16, 17, and 18 were selected for use; each of these items address potential bias associated with follow-up assessments in longitudinal studies. In addition, alignment of studies with the Guidelines for the Conduction of Follow-up Studies Measuring Injury-Related Disability was analyzed [16].

The results of all studies were tabulated in order to identify the different measures used, the methods of reporting HRQL information (e.g. summary scores), and whether any changes in HRQL over time were observed. For studies presenting HRQL summary scores, the scores could range from either 0 to 1 or 0 to 100 depending on the measurement instrument used. Two examples of generic HRQL instruments that can be used to derive a summary score are the EQ-5D and the SF-36. With respect to disability, an example of an instrument that can be used to derive a summary score is the WHODAS II [22]. For all instruments examined, lower scores were representative of worse health.

## Results

### Literature search

**Fig. 1:**
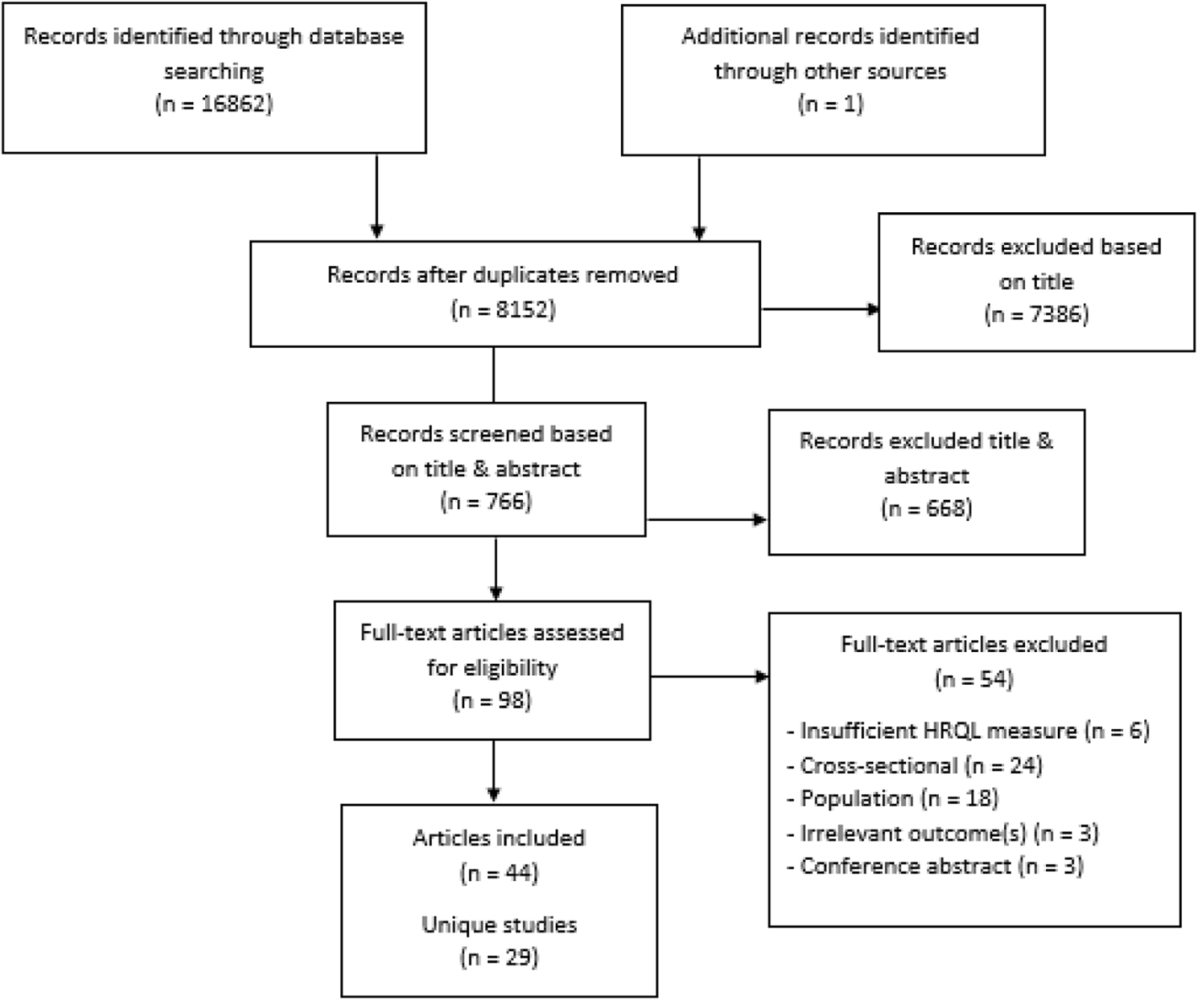
PRISMA Flow Diagram

The search strategy in the specified databases provided a total of 8152 unique potentially relevant articles (see Fig. 1). One additional article that did not turn up in our search was extracted from the reference list of an included study, and added to the relevant titles. In the first selection round, based on scanning the titles, 7386 articles were excluded. The main reasons for exclusion were that studies were not about injury or were about a specific injury type, rather than injury in general. The abstracts of the remaining 766 articles were read in the next selection round, resulting in the exclusion of 668 more articles due to a lack of HRQL measurement. The full texts of the remaining 98 articles were read, and led to the final inclusion of 44 articles. These articles represent 29 unique studies. The main reason for final exclusion of 54 articles was a lack of a sufficient HRQL measurement or the lack of multiple HRQL measurements.

### Study characteristics

Study characteristics are presented in Table 1. Out of the 44 articles that were included in our systematic review, most (*n* = 12) reported findings from a single prospective cohort study conducted in New Zealand [14, 27–37]. Seven articles were published using data from Australia [24–26, 39–42], with two articles related to the same study cohort from Victoria [41, 42] and two articles related to the same cohort from South-East Queensland [25, 26]. Five articles reported on five unique studies conducted in the United States [38, 53, 57, 64, 65]. Three articles resulted from two studies in Switzerland [43–45] and three articles resulted from two studies in Norway [59, 60, 62], respectively. Two articles from two different studies were detected from both Italy [46, 47] and Sweden [52, 56]. Remaining articles were from studies conducted in Hong Kong [55], India [48], British Colombia [58], Iran [23], Spain [50], United Kingdom [49], Thailand [63], Japan [61] and Vietnam [51] (all *n* = 1). One study was a multicentre study, conducted in both Australia and Hong Kong [54]. The sample sizes for each investigation ranged from 105 to 87,134, with the majority of the samples in the range of 105 to 668 participants (*n* = 28). Four studies measured HRQL in children and adolescents [48, 53, 57, 58], while all other studies focussed on adult populations. All studies included a non-specific injury population, with differing injury severities.

**Table 1:** Study characteristics of included articles measuring HRQL in general injury populations

Author, year, country	Study population and design	HRQL instrument	Follow up time points	Predictors of HRQL/Disability	Outcomes
Abedzadeh-Kalahroudi, 2015 [23], Iran	Hospitalised trauma patients (15-65y) (*N* = 400); Hospital; Prospective cohort study	WHODAS II	1 month 3 months	Predictors disability: age, length of hospital stay, injury to extremities	Disability: - 1 month mean: 30.3 (9.2) - 3 months mean: 18.8 (8.3) - Activity limitation: 11.3 (15.8) - Participation: 16.9 (20.2)
Aitken, 2012 [24], Australia	**Adult (≥18) patients with acute trauma (*N*** **= 212); Hospital; Prospective multicentre study**	**SF-36**	**Hospital discharge (92%)** **3 months (60%)** **6 months (59%)**	**PCS: age, body region containing most severe injury, perceived consequences of injury;** **MCS: age, gender, perceived ability to control environment predicted outcome**	**Slight improvement in HRQL from 3 to 6 months after hospital discharge, but not back at pre-injury level**
Aitken, 2014 [25], Australia	Trauma intensive care patients (adults) from one tertiary referral hospital admitted for acute injury (*N* = 123); Prospective cohort study	SF-36 Psychological status: Kessler Psychological Distress Scale (K10) and the PTSD Civilian Checklist	1 month (76%) 6 months (72%)	Not identified	HRQL outcome: - 1 months: PCS: 32.7 (10.4); MCS: 40.6 (15.7) - 6 months: PCS: 40.9 (13.2); MCS: 42.6 (14.0) Scores significantly below Australian norms both 1 and 6 months post-discharge
Aitken, 2016 [26], Australia	Trauma intensive care patients (adults) from one tertiary referral hospital admitted for injury (*N* = 123); Prospective cohort study	SF-36 Psychological status: Kessler Psychological Distress Scale (K10) and the PTSD Civilian Checklist	1 month (76%) 6 months (72%) 12 months (68%) 24 months (56%)	Non-modifiable factors linked with physical function: Optimistic perception of illness, greater self-efficacy, hospital length of stay, injury insurance	HRQL outcome: - 1 month: PCS: 32.7 (10.4); MCS: 40.6 (15.7) - 6 months: PCS: 40.9 (13.2); MCS: 42.6 (14.0) - 12 months: PCS: 42.8 (11.7); MCS: 42.4 (13.8) - 24 months: PCS: 43.7 (12.3); MCS: 44.6 (12.5) Averages remained below Australian norms at 24 months
Davie, 2018 [27], New Zealand	**Individuals (18-64y) from ACC entitlement claims register (*N*** **= 2856); Prospective cohort study**	**WHODAS II**	**3 months** **12 months** **24 months (65% with complete data)**	**Comorbidity**	**Percentage disabled:** **-3 months: No comorbidities: 37.2%** **1 comorbidity: 39.8%** **Multimorbidity: 51.9%** **- 12 months: No comorbidities: 10.6%** **1 comorbidity: 11.4%** **Multimorbidity: 27.1%** **- 24 months: No comorbidities: 8.9%** **1 comorbidities: 10.8%** **Multimorbidity: 24.6%**
Derrett, 2011 [14], New Zealand	**Individuals (18-64y) from ACC entitlement claims register (*N*** **= 2856); Prospective cohort study**	**EQ-5D + cognition;** **WHODAS II 12-item**	**3 months (59%)**	**Not identified (preliminary analysis only)**	**Worse HRQL and increased disability compared to pre-injury status**
Derrett, 2012 [28], New Zealand	**Individuals (18-64y) from ACC entitlement claims register (*N*** **= 2856); Prospective cohort study**	**WHODAS II**	**3 months (96%) (informed on pre-injury status and post injury status in one interview)**	**Associated with disability: pre-injury disability, obesity, higher injury severity (NISS > 3), female, ≥2 chronic conditions before injury, perceiving a threat of disability, lower extremity fracture**	**Non-hospitalised: disability experienced by 39% 3 months after injury** **Hospitalised: Phase disability more prevalent**
Derrett, 2013 [29], New Zealand	**Individuals (18-64y) from ACC entitlement claims register (*N*** **= 2856); Prospective cohort study**	**WHODAS II**	**24 months (76%)**	**Post-injury disability:** **- Hospitalised: WHODAS ≥ 10, ≥2 chronic conditions pre-injury, not being optimistic pre-injury, BMI ≥ 30, smoking, perceived threat of long term disability, trouble accessing health care, head/neck superficial injury, lower extremity open wound** **- Non-hospitalised: WHODAS ≥ 10, ≥2 chronic conditions pre-injury, depressive type episode pre-injury, BMI ≥ 30, smoking, intentional injury, trouble accessing health care, intracranial injury, spine sprain/dislocation**	**Disability at 24 months:** **- Hospitalised: 13.1%** **- Non-hospitalised: 13.0%** **- Māori: 19%** **- Pacific participants: 15%**
Harcombe, 2015 [30], New Zealand	**Individuals (18-64y) from ACC entitlement claims register (*N*** **= 2856); Prospective cohort study**	**EQ-5D**	**3 months** **12 months** **24 months (25–28% missing at least 1 response)**	**Not identified**	**Attain pre-injury status:** **- Hospitalised:** **3 months: 20%** **12 months: 28%** **24 months: 34%** **- Non-hospitalised:** **3 months: 30%** **12 months: 35%** **24 months: 36%**
Langley, 2013 [31], New Zealand	**Individuals (18-64y) from ACC entitlement claims register (*N*** **= 2856); Prospective cohort study**	**EQ-5D + cognition**	**3 months** **12 months (80%)**	**Preinjury EQ-5D status, female, age 45–64, inadequate household income, preinjury disability, 2 or more prior chronic illnesses, smoking regularly, dislocation/sprains to spine or upper extremities, having relatively severe injury**	**Continued adverse outcomes (pain/discomfort) 12 months after injury**
Maclennan, 2013 [32], New Zealand	**Individuals of Māori ethnicity from ACC entitlement claims register (18-64y) (*N*** **= 566); Prospective cohort study**	**EQ-5D + cognition;** **WHODAS II 12-item**	**3 months (59%)**	**Not identified**	**HRQL:** **- Walking difficulties: +/− half cohort** **- Pain/discomfort: 2/3 of cohort** **- Psychological distress: > 1/2 cohort** **- Disability: 49%** **- Satisfied with life: majority** **- Consider themselves in good/excellent health: majority**
Maclennan, 2014 [33], New Zealand	**Individuals (18-64y) from ACC entitlement claims register (*N*** **= 2856); Prospective cohort study**	**WHODAS II & EQ-5D + cognition**	**3 months** **12 months (80%)**	**Not identified**	**Pre-injury:** **- Non-Māori: > 90% good health** **- Māori: > 90% good health** **12 months:** **- Non-Māori: problems increased 4–40%** **- Māori: problems increased 5–45%**
Mauiliu, 2013 [34], New Zealand	**Individuals (18-64y) from ACC entitlement claims register (*N*** **= 2856); Prospective cohort study**	**EQ-5D** **WHODAS II**	**3 months (59%)**	**Less likely to have problems with disability & HRQL: Pacific people**	**Pacific people less likely to have:** **- Disability: no/lesser problems** **- Self-care: no problems** **- Anxiety/depression: no problems**
Wilson, 2013 [35], New Zealand	**Individuals (18-64y) from ACC entitlement claims register (*N*** **= 2856); Prospective cohort study**	**EQ-5D + cognition**	**12 months (78%)**	**Sex, injury severity, hospitalisation status**	**Mean QALYs lost first year after injury:** **- Male: 0.21 QALY** **- Female: 0.24 QALY** **- Hospitalised: 0.25 QALY** **- Non-hospitalised: 0.21 QALY**
Wyeth, 2017 [36], New Zealand	**Individuals (18-64y) from ACC entitlement claims register (*N*** **= 2856); Prospective cohort study**	**WHODAS II**	**3 months** **24 months (66%)**	**Disability at 24 months: ≥2 chronic conditions pre-injury, trouble accessing healthcare services after injury; hospitalisation for injury, inadequate pre-injury household income**	**Percent disability:** **- Pre-injury: 9%** **- 24 months: 19%** **- Age 30–49: 23% (highest proportion)**
Wyeth, 2018 [37], New Zealand	**Individuals (18-64y) from ACC entitlement claims register (*N*** **= 2856); Prospective cohort study**	**WHODAS**	**24 months (80% non-Māori; 66% Māori)**	**Māori: not working for pay before injury, experiencing disability before injury, trouble accessing healthcare services for injury** **Non-Māori: inadequate household income prior to injury, less than secondary school qualifications, not working for pay, disability prior to injury, ≥2 chronic conditions, BMI ≥ 30**	**RR of disability 24 months after injury:** **Māori:** **- Hospitalised, non-working: 2.7 (1.4, 4.9)** **- Pre-injury disabled: 3.1 (1.6, 5.8)** **- Difficulties accessing health care: 2.6 (1.3, 5.2)** **Non-Māori:** **- Hospitalised, inadequate household income: 2.4 (1.4, 4.1)** **- Less than secondary school qualification: 2.0 (1.1, 3.8)** **- Not working for pay before injury: 2.8 (1.5, 5.1)** **- Disability before injury: 3.0 (1.7, 5.2)** **- ≥ 2 chronic conditions: 3.5 (2.0, 6.4)** **- BMI ≥ 30: 2.4 (1.3, 4.4.)**
Dhungel, 2015 [38], US	Adult (18+) trauma population divided in groups of normal weight, overweight, obese and morbidly obese (*N* = 235); Trauma centre; Prospective cohort study	FIM	Admission Hospital discharge 6 months (79%)	Not defined	Functional Status: - Admission: Non-obese: 38.2 (13.9) Overweight: 40.0 (11.1) Obese: 38.3 (15.1) Morbidly obese: 41.6 (13.9) - Discharge: Non-obese: 62.4 (7.9) Overweight: 60.0 (8.4) Obese: 56.7 (13.0) Morbidly obese: 58.7 (9.3) - Follow-up: Non-obese: 71.1 (2.1) Overweight: 70.6 (3.4) Obese: 70.3 (3.8) Morbidly obese: 69.8 (5.4)
Dinh, 2016 [39], Australia	**Adult (≥16) trauma patients (*N*** **= 349); Major trauma centre; Prospective cohort study**	**EQ-5D and SF-12**	**Baseline** **3 months** **6 months (51%)**	**Physical health: lower limb injuries; Mental health: mechanism of injury, past mental health; RTW: increasing ISS, upper limb injuries**	**HRQL: No significant change in PCS and MCS between 3 and 6 months**
Gabbe, 2013 [40], Australia	Adult major trauma patients (*N* = 662); Level 1 trauma centre; Prospective cohort study	SF-12 GOSE	6 months 12 months 18 months 24 months (93% followed up for at least 1 time point)	Not defined	- 6-12 months: Functional recovery, RTW, physical health improved - > 12 months: little change - < 18 months: mental health score decreased - 18-24 months: mental health score improved
Gabbe, 2016 [41], Australia	**Adult major trauma survivors (*N*** **= 8844); Victorian State Trauma Registry (VSTR); Prospective cohort study**	**GOS** **GOSE**	**6 months** **12 months** **24 months (74% for all follow-up points)**	**Female, older patients, pre-existing conditions, spinal cord injured and multi-trauma patients involving head injury, intentional/low-fall events, compensable patients, greater socioeconomic disadvantage, pre-existing drug/alcohol/mental health conditions**	**Good recovery:** **- 6 months: Male: 33.2%; Female: 27.2%** **- 12 months: Male: 37.3%; Female: 28.8%** **- 24 months: Male: 39.7%; Female: 31.1%**
Gabbe, 2017 [42], Australia	**Hospitalised adult major trauma patients (ISS ≥ 12) (*N*** **= 2424); Victorian State Trauma Registry (VSTR); Prospective cohort study**	**EQ-5D-3 L**	**6 months (84%)** **12 months (85%)** **24 months (84%)** **36 months (74%)**	**Age, compensable status, level of education, nature of injuries, gender, preinjury employment, level of socioeconomic disadvantage**	**HRQL:- 6 months: 0.67 (0.31)** **- 12 months: 0.68 (0.32)** **- 24 months: 0.71 (0.31)** **- 36 months: 0.70 (0.32)**
Gross, 2011 [43], Switzerland	Patients treated primarily at a university trauma centre after blunt polytrauma (*N* = 178); University hospital ICU; Prospective cohort study	EQ-5D SF-36 MFA TOP	24 months (57%)	Long term pain associated with HRQL-scores	Mean (SD) HRQL: EQ-5D pain: - Pre-injury: 1.1 (0.4) - Post-injury: 1.7 (0.6) SF-36 pain: - Pre-injury: 94.3 (14.1) - Post injury: 65.0 (29.5) MFA pain: - Pre-injury: 1.4 (0.7) - Post-injury: 2.4 (1.2) TOP total pain: - Pre-injury: 96.2 (7.7) - Post injury: 72.0 (29.7)
Gross, 2012 [44], Switzerland	Polytrauma patients defined as trauma victims with ISS ≥ 16 (*N* = 170); University hospital ICU; Prospective cohort study	EQ-5D SF-36	2.5 years (65%)	Negative association with EQ-5D and SF-36: Brain injury	HRQL: EQ-VAS: - Pre-injury: Non-TBI: 88.5 (17.6); TBI: 91.4 (9.5) - Post-injury: Non-TBI: 69.9 (23.4); TBI: 59.4 (25.0) EQ-5D: - Pre-injury: Non-TBI: 94.5 (13.7); TBI: 98.6 (3.6) - Post-injury: Non-TBI: 76.4 (20.8); TBI: 65.4 (27.7) SF-36: - Pre-injury: PCS: non-TBI: 56.0 (6.9); TBI: 56.8 (5.5) MCS: non-TBI: 50.8 (11.8); TBI: 50.3 (11.3) - Post-injury: PCS: non-TBI: 45.3 (10.6); TBI: 44.0 (11.9) MCS: non-TBI: 48.1 (12.9); TBI: 38.9 (13.1)
Gross, 2019 [45], Switzerland	**Major trauma patients (15-63y) (NISS ≥ 8) (*N*** **= 1078); Teaching hospital; Prospective cohort study**	**SF-36, EQ-5D & GOS**	**1 year** **2 years** **(31.2% year 1 & 2)**	**Associated with GOS outcomes between 1-2y after trauma: gender, age, trauma, energy, length of hospital stay**	**HRQL:** **EQ-5D:** **- 1 year:** **Male: 0.74 (0.22)** **Female: 0.77 (0.19)** **- 2 years:** **Male: 0.74 (0.22)** **Female: 0.80 (0.15)** **SF-36:** **- 1 year:** **Male: PCS: 46.11 (9.78); MCS: 49.25 (12.66)** **Female: PCS: 47.54 (9.24); MCS: 47.92 (11.81)** **- 2 years:** **Male: PCS: 46.29 (9.97); MCS: 50.14 (12.78)** **Female: PCS: 48 .8(8.18); MCS: 49.61 (10.60)**
Innocenti, 2014 [46], Italy	Adult (≥18) patients admitted in ED-HDU for trauma (*N* = 418); Prospective cohort study	SF-12	6 months (58%)	Not defined	Pre-injury: - MCS: normal score: 94% - PCS: normal score: 96% After injury: - MCS: normal score: 70% - PCS: normal score: 58%
Innocenti, 2015 [47], Italy	**Mild to moderate trauma patients admitted to ED high dependency unit (*N*** **= 286); Prospective cohort study**	**SF-12**	**6 months (53%)**	**Older age, female, pre-existing medical conditions, high Sequential Organ Failure Assessment score**	**Pre-injury:** **- PCS: 53 (7)** **- MCS: 55 (7)** **6 months:** **- PCS: 41 (12)** **- MCS: 46 (13)** **Maintain normal value after injury:** **PCS: 52%** **MCS: 68%**
Jagnoor, 2017 [48], India	Children (2-16y) with overnight admission to hospital due to injury (*N* = 386); Hospital/secondary/tertiary care institution; Prospective multicentre study	PedsQL	Pre-injury (97%) 1 month (73%) 2 months 4 months 12 months (77% all time points)	Not defined	Mean score: - Baseline: Physical score: 99.4 (3.4) Psychosocial score: 99.4 (3.4) - 1 month: Physical: 79.7 Psychosocial: 86.3 - 2 months: all scores improved
Kendrick, 2017 [49], UK	**Patients (16-70y) with unintentional injury that required hospital admission (*N*** **= 668); Hospital; Prospective multicentre study**	**EQ-5D-3 L**	**1 month (77%)** **2 months (72%)** **4 months (68%)** **12 months (63%)**	**Associated with clinically important reductions in HRQL between 2 & 12 months post-injury: Higher depression and anxiety scores**	**HRQL:** **- Pre-injury: 0.92 (0.18)** **- 1 month: 0.44 (0.28)** **-2 months: 0.57 (0.27)** **- 4 months: 0.69 (0.23)** **- 12 months: 0.78 (0.21)** **60% respondents 12 months after injury lower HRQL than pre-injury**
Llaquet, 2018 [50], Spain	Injured adult (≥16) patients admitted to intensive care unit in Spanish level 1 trauma centre (*N* = 304); Prospective cohort study	EQ-5D-5 L	Hospital discharge 3 months 6 months 12 months (66%)	Lower EQ-VAS: Age ≥ 55, female, unskilled employment	HRQL: EQ-VAS: - Discharge: 60 - 3 months: 65 - 6 months: 70 - 12 months: 75
Nguyen, 2018 [51], Vietnam	**Adult injury patients hospitalised for at least 1 day (*N*** **= 892); Hospital; Prospective cohort study**	**HUI3**	**1 month (86%)** **2 months (86%)** **4 months (85%)** **12 months (82%)**	**Older age, more severe injury, other illnesses**	**HRQL:** **- 1 month: Males: 0.52; Female: 0.28** **- 2 months: Males: 0.67; Females: 0.47 l** **-4 months: Males: 0.77; Females: 0.57** **- 12 months: Males: 0.87; Females: 0.71**
Orwelius, 2012 [52], Sweden	Adult patients with emergency admission to ICU (*N* = 146); ICU; Prospective multicentre study	SF-36	6 months (74%) 12 months (58%) 24 months (39%)	Associated with HRQL: Pre-existing disease, Maximum SOFA score, APACHE-II score, marital status	- 6-12 months: significant improvements for role limitations caused by physical problems; improvement in bodily pain - 12-24 months: further improvements
Pieper, 2015 [53], US	**Children 8–17 with mild (brain) injury or no injury (*N*** **= 120); Paediatric emergency department; Prospective cohort study**	**PedsQL**	**Baseline (preinjury)** **1 month** **3 months** **6 months** **12 months (86%)**	**Not defined**	**Total generic health:** **- Baseline: Child: 83.5 Parent: 86.9** **- 1 month: Child: 83.1 Parent: 84.2** **- 3 months: Child: 86.1 Parent: 85.6** **- 6 months: Child: 87.4 Parent: 85.7** **- 12 months: Child: 88.6 Parent: 87.0**
Rainer, 2014 [54], Hong Kong/Australia	Adult (≥18) Major trauma patients (ISS ≥ 16); (Hong Kong: *N* = 225; Australia: *N* = 1752); Trauma registry; Prospective multicentre study	SF-12 GOSE	6 months (HK: 72.4%; Australia: 83.4%) 12 months (HK: 62.1%; Australia: 85.8%)	Sex, age, ISS, Glasgow Coma Scale	PCS: - 6 months: HK: 42.7 (9.8); AUS: 41.6 (11.8) - 12 months: HK: 42.2 (11.0); AUS: 42.6 (12.0) MCS: - 6 months: HK: 51.8 (12.4); AUS: 50.6 (11.4) - 12 months: HK: 52.2 (10.9); AUS: 50.3 (11.2)
Rainer, 2014 [55], Hong Kong	**Adult (≥18) patients moderate/major trauma (ISS ≥ 9) (*N*** **= 400); Prospective multicentre study**	**SF-36** **GOSE**	**Baseline (preinjury) Discharge-30 days (84%)6 months (70%) 12 months (59%)**	**Age > 65, male, pre-injury health problems, admission to ICU, ISS, baseline, 1 and 6 month PCS, 6 month MCS (univariate analysis only)**	**GOSE: Upper good recovery %:** **- Baseline: 3.5%** **- 1 month: 9.7%** **- 6 months: 16.0%** **- 12 months: 16.5%** **HRQL: % above norm** **PCS: (norm HK: 52.83)** **- Baseline: 4.8%** **- 1 month: 6.7%** **- 6 months: 15.0%** **- 12 months: 15.5%** **MCS: (norm HK: 47.18)** **- Baseline: 57.0%** **- 1 month: 28.5%** **- 6 months: 39.7%** **- 12 months: 31.2%**
Ringdal, 2010 [56], Sweden	Adult injury patients that required intensive care (*N* = 344); Hospital; Prospective multicentre study	SF-36	4.5y to 5.5y after injury (71%)	Delusional memories during ICU stay, pre-existing disease prior trauma	0.5–1.5 years: - PCS: 65.9 (31.6) - MCS: 63.7 (27.3) 4.5–5.5 years: - PCS: 71.9 (30.1) - MCS: 71.2 (22.5)
Rivara, 2014 [57], US	**Trauma patients (parents & children), with only parent injured, only child injured, both injured or neither injured (*N*** **= 570); Medical Centre; Prospective cohort study**	**SF-36 (injured)** **SF-12 (non-injured)**	**5 months** **12 months (34%)**	**Parents injury affects child HRQL**	**Baseline HRQL:** **PCS:** **Both injured: 55.5 (9.4)** **Child injured: 52.0 (8.2)** **Parent injured: 54.8 (9.1)** **Neither injured: 53.2 (8.5)** **MCS:** **Both injured: 55.3 (8.3)** **Child injured: 51.6 (7.9)** **Parent injured: 54.0 (9.0)** **Neither injured: 49.9 (11.2)**
Schneeberg, 2016 [58], British Columbia	Children (0-16y) who presented with primary injury at British Columbia Children’s Hospital (*N* = 582); Prospective cohort study	PedsQL 4.0 Generic Core PedsQL infant scales	Pre-injury (+ at least 1 follow-up: 35%) 1 month (44%) 4–6 months (29%) 12 months (28%)	Greater impact on HRQL 1 month post injury, steeper slope to recovery: Older age, hospitalisation	Mean HRQL: - Baseline: 90.7 - 1 month: 77.8 - 4 months: 90.3 - 12 months: 91.3
Soberg, 2012 [59], Norway	**Patients 18-67y with an NISS ≥ 16 and at least 2 injuries classified in AIS (*N*** **= 105); University hospital; Prospective cohort study**	**SF-36** **WHODAS II**	**6 weeks** **1 year (99%)** **2 years (94%)** **5 years (80%)**	**PCS: Time points of measurement, time in hospital/rehabilitation, getting around, participation in society** **MCS: time points of measurement, sex, education, WHODAS II cognitive function & participation in society**	**WHODAS-II scores:** **Understanding/communicating:** **- 6 weeks: 10.0 (0.0–30.0)** **- 1 year: 10.0 (0.0–25.0)** **- 2 years: 10 (0.0–25.0)** **- 5 years: 10.0 (0.0–30.0)** **Getting around:** **- 6 weeks: 37.5 (12.5–62.5)** **- 1 year: 12.5 (0.0–37.5)** **- 2 years: 12.5 (0.0–37.5)** **- 5 years: 12.5 (0.0–31.3)** **Self-care:** **- 6 weeks: 20.0 (0.0–30.0)** **- 1 year: 0.0 (0.0–10.0)** **- 2 years: 0.0 (0.0–10.0)** **- 5 years: 0.0 (0.0–10.0)** **Getting along with people:** **- 6 weeks: 16.7 (8.3–35.4)** **- 1 year: 16.7 (0.0–25.0)** **- 2 years: 16.7 (8.3–33.3)** **- 5 years: 20.8 (8.3–33.3)** **Life activities:** **- 6 weeks: 50.0 (35.0–80.0)** **- 1 year: 30.0 (10.0–50.0)** **- 2 years: 40.0 (0.0–50.0)** **- 5 years: 20.0 (0.0, 50.0)** **Participation in society:** **- 6 weeks: 45.8 (37.5–58.3)** **- 1 year: 25.0 (12.5–41.7)** **- 2 years: 25.0 (8.3–41.7)** **- 5 years: 18.8 (8.3–34.4)**
Soberg, 2015 [60], Norway	**Patients (18-67y) with severe multiple injuries (*N*** **= 105); Hospital; Prospective cohort study**	**SF-36**	**1 year** **2 years** **5 years** **10 years (55.2%)**	**PCS: change in coping from 2 to 10 years** **PCS and MCS: bodily pain at 2 years;** **MCS: change in coping, vitality at 1 year, social functioning and mental health at 2 years**	**10 years:** **- PCS: 41.8 (11.7)** **- MCS: 48.8 (10.7)** **Reduced PCS compared with adjusted general population; MCS not different from general population**
Tamura, 2018 [61], Japan	All eligible consecutive trauma patients admitted to the intensive care unit of one tertiary care hospital (*N* = 187); Prospective cohort study	SF-36	6 months (84%) 12 months (69%)	Not identified	Median [IQR]: - Discharge: PCS: 21 [10, 35]; MCS: 56 [48, 66] - 6 months: PCS: 43 [33,51]; MCS: 52 [44, 61] - 12 months: PCS: 44 [32, 53]; MCS: 53 [46, 59] Role Social: - Discharge: 21 [10, 38] - 6 months: 39 [23, 52] - 12 months: 45 [29, 53] 12 months post injury: 12% dependent on home care
Tøien, 2011 [62], Norway	**Hospitalised trauma patients (18-75y) (*N*** **= 393); Trauma referral centre; Prospective cohort study**	**SF-36**	**3 months (77%)** **12 months (64%)**	**All dimensions: optimism; Physical functioning: high depression score baseline, lower age, head injury; Mental functioning: high depression score baseline, higher age, being employed or studying before trauma; Bodily pain & vitality: high depression score baseline; General health: optimism, low PTSD at baseline, lower ISS**	**HRQL: differences men/women** **3 months:** **- Mental health: Men: 76.6; Women: 71.3** **- Vitality: Men: 57.3; Women: 46.6** **12 months:** **- Vitality: Men: 56.8; Women: 50.0**
Yiengprugsawan, 2014 [63], Thailand	Distance learning students 15-87y enrolled at Sukhothai Thammathirat Open University (*N* = 87,134); Prospective cohort study	MOS-SF-8	4 years (70%)	Injury exposure	HRQL injury yes/no: PCS: - 2005-no 2009-no: 50.2 [49.8–50.5] - 2005-yes 2009-no: 47.4 [46.3–48.4] - 2005-no 2009-yes: 49.2 [48.3–50.1] - 2005-yes 2009-yes: 46.3 [44.6–48.1] MCS: - 2005-no 2009-no: 48.0 [47.6–48.4] - 2005-yes 2009-no: 46.0 [44.8–47.2] - 2005-no 2009-yes: 47.1 [46.0–48.2] - 2005-yes 2009-yes: 44.9 [42.8–46.8]
Zarzaur, 2016 [64], US	**Traumatically injured adult patients (≥18) (*N*** **= 500); Trauma centre; Prospective cohort study**	**SF-36**	**1 month (93%)** **2 months (82%)** **4 months (70%)** **12 months (58%)**	**3 PCS trajectories, 5 MCS trajectories:** **PCS: 1. Low baseline score, no improvement; 2. Declines 1 month after injury, then improves over time; 3.Sharp decline followed by rapid recovery; MCS 1. Low baseline, remain low; 2. Large decrease post-injury, no recovery over next 12 months; 3.initial decrease in MCS early, followed by continuous recovery; 4. Steady decline over study period; 5. Consistently high at all time points**	**Not identified**
Zarzaur, 2017 [65], US	Traumatically injured patients (≥18y) (*N* = 225); Level 1 trauma centre; Prospective cohort study	SF-36	Baseline (preinjury) 1 month (94%) 2 months (83%) 4 months (69%) 12 months (64%)	PCS: individual income; MCS: high resiliency score; age; income	Different trajectories of recovery - Either improvement of physical and/or mental health or decline

*ISS* Injury Severity Score, *SF-12* Medical Outcome Study Short Form-12 items, *GOSE* Extended Glasgow Outcome Scale, *SF-36* Medical Outcome Study Short Form-36 items, *ICU* Intensive Care Unit, *PCS* Physical Component Score, *MCS* Mental Component Score, *EQ-5D-3 L* EQ-5D with three response options per dimension, *GOS* Glasgow Outcome Scale, *HUI3* Health Utilities Index 3, *PTSD* Post-traumatic Stress Disorder, *MOS-SF-8* Medical Outcome Study Short-Form, *ED* Emergency Department, *ACC* Accident Compensation Corporation, *QALY* Quality Adjusted Life Year, *WHODAS II* World Health Organization Disability Assessment Schedule version II, *BMI* Body Mass Index, *NISS* New Injury Severity Score, *MFA* Musculoskeletal Functional Assessment, *TOP* Trauma Outcome Profile, *AIS* Abbreviated Injury Scale, *SOFA* Sequential Organ Failure Assessment, *APACHE-II* Acute Physiology Age Chronic Health Evaluation, *ED-HDU* Emergency Department High Dependency Unit, *RTW* Return To Work, *EQ-VAS* European Quality of Life instrument Visual Analogue Scale, FIM Functional Independence Measure, *PedsQL* Paediatric Quality of Life Inventory Generic Core Scales

Articles are ordered alphabetically, and articles that come one after the other and have the same bold/non-bold font are from the same study

Approximately a third (*n* = 10) of all studies focused on all injury severities, with a main inclusion criteria of hospital admission or injuries likely to result in insurance claims for more than just medical treatment. The second largest group of studies focussed on major injuries (*n* = 18). Inclusion criteria were varying, with some studies only requiring ≥24 h stay at the hospital or admission to intensive care unit (ICU) (*n* = 7), and other studies requiring a minimum score on the ISS (Injury Severity Score) or NISS (New Injury Severity Score). ISS for major injuries ranged from ISS > 12 (*n* = 2) to ISS ≥16 (*n* = 2), versus NISS ranging from NISS ≥8 (*n* = 1) to NISS ≥16 (n = 2). The remaining 5 studies focused on moderate (*n* = 3) or mild to moderate (*n* = 2) injuries, with moderate injury studies requiring AIS (Abbreviated Injury Scale) ≥2 (*n* = 1) or ISS ≥9 (*n* = 2), and mild to moderate injury studies requiring ISS < 15 (*n* = 1) and length of hospitalisation < 24 h (*n* = 1).

### Study design

**Fig. 2:**
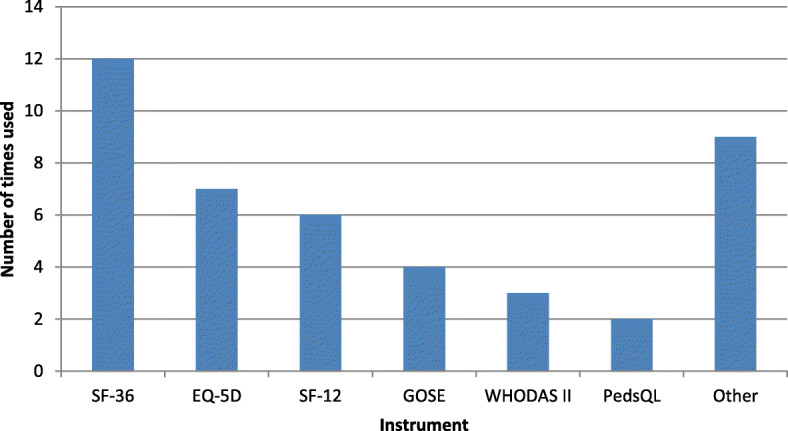
Frequency of generic measures used in studies to assess HRQL. Note1: Some studies used more than 1 measurement instrument. Note2: ‘Other’ consists of: GOS (2), HUI3 (1), MOS-SF-8 (1), MFA (1), TOP (1), FIM (1), PedsQL 4.0 Generic core (1), PedsQL infant scales (1)

**Fig. 3:**
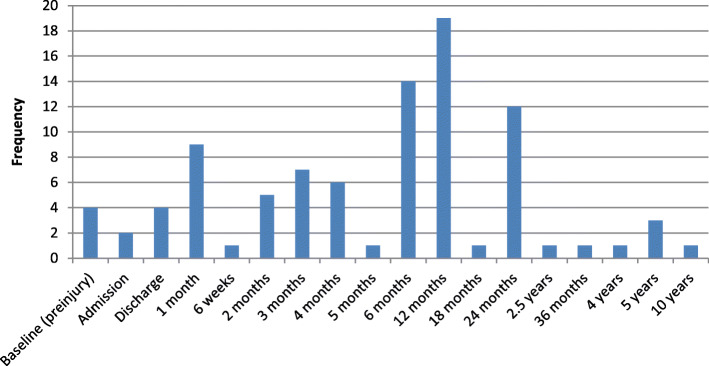
Frequency of time points at which HRQL was measured across studies

All studies that were included in this review were prospective cohort studies. Seven out of the 29 unique studies were multicentre studies [24, 48, 49, 52, 54–56]. Across studies HRQL and disability were measured with 14 different measurement instruments. Generic instruments SF-36 (*n* = 13) and EQ-5D (*n* = 7) were most commonly used, followed by SF-12 (*n* = 6) and GOSE (*n* = 4), as can be retrieved from Fig. 2. Approximately 45% of the studies (*n* = 13) used more than one measurement instrument, of which 10 used two instruments, and 3 used more than two instruments. All measurement instruments were generic, with three out of four studies in children using a child-specific instrument (PedsQL; PedsQL 4.0; PedsQL infant scales) only, and one study in children using two all ages instruments (SF-12 and SF-36). Measurement of HRQL was conducted at different time points in studies, with the number of follow-up points varying from one (*n* = 4) to five (*n* = 3). HRQL was assessed at more than one follow-up point in 25 studies, with measurement at 6 and 12 months most frequent across all studies (*n* = 14 and *n* = 19, respectively) (Fig. 3). Three other common measurement points were 24 months (*n* = 12), 1 month (*n* = 9) and 3 months (*n* = 7) after injury. Studies used different administration methods of questionnaires, with telephone interview as the most common method (*n* = 13). A combination of different methods was common, with baseline measurement often performed in a face-to-face interview, and later follow-up measurements done by either telephone or postal/email interview.

### Quality of studies

Length of follow-up was consistent for all study participants in all but two studies [25, 26, 56]. The same results were found regarding whether follow-up time was sufficient for measuring primary outcomes, with only two studies reporting an insufficient follow-up period [24, 47]. However, attrition appeared to be a problem in many studies: 18 out of 29 studies exceeded the attrition norm of 20% for < 1 year follow-up and 30% for ≥1 year follow-up.

Regarding adherence to the Guidelines for the Conduction of Follow-up Studies Measuring Injury-Related Disability, it was found that study populations were generally in accordance with the guidelines. However, measurement in respondents with mental and/or social problems was only specifically mentioned in two studies [40, 48], whereas all other studies provided no or unclear information on the subject. Even though the guidelines recommend a combination of the EQ-5D and HUI3 to measure HRQL, none of the included studies used this combination. The EQ-5D and HUI3 were used separately in a number of studies [14, 30–35, 39, 42–45, 49–51]. Six studies complied to the measurement points required by the guidelines, namely one, two, four and 12 months after injury [48, 49, 51, 58, 64, 65]. Even though other studies did not follow all required measurement points, the majority complied with at least one.

### Predictors for HRQL

Recovery patterns of HRQL after injury were found to differ across subgroups in most studies. There was substantial variation in the predictors of HRQL after injury, however, seven predictors were mentioned in six or more articles: age (*n* = 14), gender (*n* = 12), pre-injury health status (*n* = 12), hospitalisation status (*n* = 7), nature of injury (*n* = 7), injury severity (*n* = 7) and socio-economic status (*n* = 6). Older age and female gender were found to have a negative impact on the outcome of HRQL after trauma in several articles [24, 31, 41, 47, 50, 51], whereas in two other articles male gender was found to have a negative association with HRQL [45, 55].

### Changes over time

**Fig. 4:**
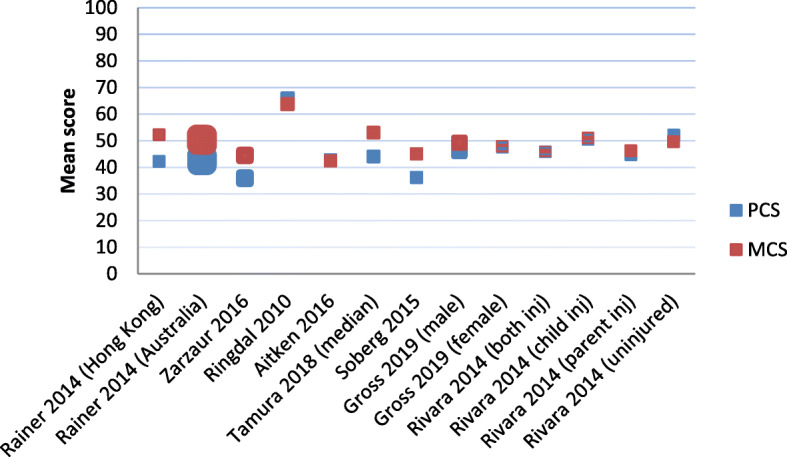
SF-12 and SF-36 scores at 12 months after injury. Note1: The y-axis shows the mean scores, not utility values. Note2: The size of the dots is proportional to the sample size

**Fig. 5:**
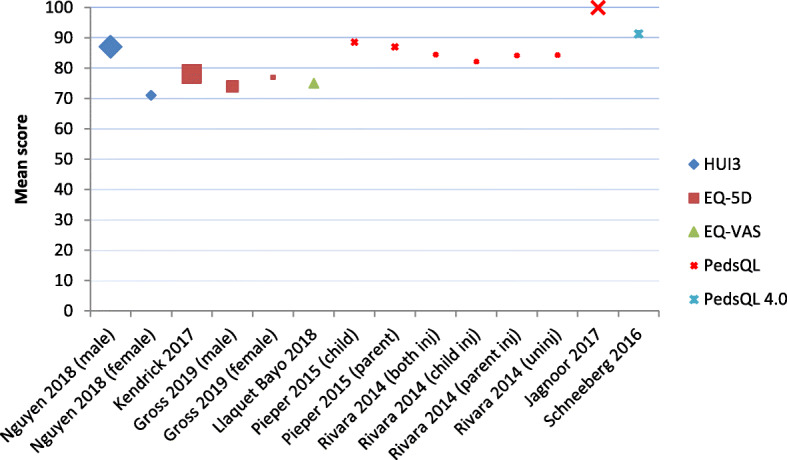
EQ-5D, PedsQL (4.0), HUI3 and EQ-VAS scores at 12 months after injury. Note1: The y-axis shows descriptive summary scores only, not utility values. Scores are not directly comparable due to the different HRQL measures used. Note2: Scale from 0 to 100 for PedsQL (4.0) and EQ-VAS; scale from 0 to 1 for EQ-5D and HUI3 (score multiplied by 100). Note3: The size of the dots is proportional to the sample size of the study

Studies that reported HRQL values generally reported improvements in HRQL over time (see Table 1). However, not all studies that were included reported specific outcomes of HRQL, as some studies reported on odds ratio and relative risks. Improvement in HRQL was found in all studies, however, pre-injury status or population level was not reached for the total injury population after 6–24 months [24, 26, 31, 36, 44, 46, 47, 49, 55, 60, 62]. Figures 4 and 5 summarise HRQL scores of all articles that provided a mean HRQL score at 12 months after injury. Some articles provided mean scores only per subgroup, and have therefore been included in the figure for each subgroup. Figure 4 shows the physical component score (PCS) and mental component score (MCS) for both SF-12 and SF-36, whereas Fig. 5 shows the summary score for the EQ-5D, EQ-VAS, HUI3 and PedsQL (4.0).

## Discussion

This systematic review aimed to provide an update on studies measuring HRQL with a generic instrument in general injury populations since the publication of an earlier review examining injury studies conducted between 1995 and 2009 [12]. Given the increase in the number of studies conducted in this area over recent years, our review focused specifically on studies that examined HRQL at more than one time point. As with the earlier review, considerable methodological variation across studies was found; differences were apparent in study settings, injury severity of participants, HRQL instruments used, follow-up periods, and timing of HRQL assessments. The most commonly used instruments to assess HRQL included the SF-36, SF-12, and EQ-5D, although 14 different instruments were applied across the 29 studies included in this review. Study follow-up points ranged from 1 month to 10 years post-injury, with follow-up assessments most commonly occurring at 6, 12 and 24 months after injury.

Despite the variation across studies included in this review, it is important to note that improvement in the consistency of study designs was observed since the earlier review of studies measuring HRQL in general injury populations [12]. Our review found a greater number of studies that had employed a longitudinal design over a shorter review period; we identified 29 longitudinal studies over a 9 year period in contrast to the 21 longitudinal studies published across the 14 years examined by Polinder et al. Our updated review also found that longer durations of follow-up have been utilised, with four studies examining HRQL beyond 24 months, and one up to 10 years post-injury. This is in contrast to the earlier review where many studies had examined outcomes until 6 months only, and none had examined outcomes beyond 24 months. These findings demonstrate an increase in adherence to the recommendations of the European Consumer Safety Association [16], which recommends assessments be conducted to a minimum of 12 months post-injury.

While longer follow-up periods are occurring in studies examining HRQL in general injury populations, the timing of assessments continues to vary across studies. The 2007 guidelines recommend assessments at regular intervals of 1, 2, 4 and 12 months post-injury, allowing for examination of the four phases of trauma recovery: acute treatment phase, rehabilitation phase, adaptation phase, and stable end situation [16]. Only five studies completed follow-ups at these time points [48, 49, 51, 64, 65], although five completed assessments at four different times in the 12 months after injury [50, 53, 58], and five examined outcomes at least four times over a longer period (beyond 12 months) [26, 40, 42]. There may be important reasons for researchers selecting different times of outcome assessment than those recommended. For example, examination beyond the 12 month point is likely to be important given accumulating evidence that changes (including improvements and deteriorations) in health status can continue to be detected after this time [59, 60]. Ensuring that participant burden is kept to a minimum is likely to be another important consideration.

Guidelines for the examination of health status among injury populations also recommend the inclusion of a retrospective recalled assessment of pre-injury health [16, 66]. Few studies in our review met this criteria, despite evidence that such retrospective measurements are likely to be more appropriate than comparisons with general population norms when evaluating post-injury losses in HRQL [9, 67]. This is because individuals from the general population are unlikely to be representative of those from an injured population [68]. A systematic review of studies collecting pre-injury HRQL data among injury patients has demonstrated that both general population comparisons and retrospective assessments are likely to result in biased estimates of pre-injury HRQL [69]. However, prospective HRQL data is often impractical to collect prior to an injury occurring. Instead, it may be most feasible to collect retrospective assessments of pre-injury HRQL as soon as practicably possible after injury.

The identification of 14 different instruments to evaluate HRQL across the 29 studies included in this updated review suggests that there remains significant variation in the types of measures used. However, it is important to recognise that this variation has decreased substantially since the earlier systematic review of studies evaluating HRQL after injury, from which 24 different generic HRQL and functional status measures were extracted. This indicates that the potential to make comparisons across studies is increasing. While a number of studies employed the EQ-5D in isolation, no studies used both the EQ-5D and the HUI3 to evaluate HRQL, which is recommended in the guidelines [16]. Many studies used neither the EQ-5D nor the HUI3, instead employing the SF-12 or SF-36 to assess HRQL. Understanding motivations behind the selection of instruments to examine HRQL and disability outcomes after injury is an important avenue for future research. Different outcome measures focus more or less on specific HRQL dimensions and the dimensions of interest to researchers may vary across countries depending on the aspects of health that are most relevant to each unique social, cultural, and political context.

Included studies varied in the reporting of HRQL information. While some studies reported the proportion of people experiencing problems with particular HRQL and disability domains others reported summary or utility scores. The 14 studies included in the review reporting summary scores represents only a slight increase from the 12 studies that did so in the earlier review.

As with the earlier review, our review found that generic instruments are capable of detecting changes in HRQL between discharge and follow-up. Despite continuing variation in study design, it is evident that the greatest gains in health status are observed in the first 12 months after injury. Gains can also be observed in the following 12 months (up to 24 months post-injury) among individuals who have sustained serious injuries (as indicated by injury severity scores and hospitalisation status). Although these gains can be detected, many studies concluded that HRQL remains significantly reduced in comparison to pre-injury levels or population norms, and this is evident up to 10 years after injury [60]. While these insights are important, continued variation in assessment time points, study populations, HRQL instruments, and the reporting of HRQL outcomes makes it difficult to compare findings from individual studies, and reduces the precision of knowledge regarding the global impact of injury on population health over time.

An important limitation associated with this systematic review is that only peer-reviewed published literature was included. It is possible that other longitudinal studies examining HRQL in large injury populations have been conducted but not published. Another limitation is that studies that examined HRQL *or* disability were eligible for inclusion in the review, and although these constructs are related, they are not synonymous. Despite these limitations, the review provides important insight into the design and findings of studies published since 2010. The variation observed across included studies suggests that the European Consumer Safety Association guidelines for the conduction of follow-up studies may be difficult for researchers to adhere to. Further research is needed to explore the reasons why researchers are not following these guidelines. This information could be used to inform the development of updated guidelines that are feasible to follow when taking into account the significant contextual variation that exists across different countries and populations. This, in turn, may lead to increased consistency in study designs and outcome reporting, allowing for meaningful cross-country comparisons.

## Conclusions

Although increased consistency in studies designed to investigate HRQL in general injury populations has been observed since 2010, there remains significant variation that makes comparisons across studies difficult and prevents precise estimates of the impact of injury on global health. Exploring reasons for variation in study design and reporting of outcomes is an important avenue for future research that may inform the development of updated guidelines for the conduct of follow-up studies measuring HRQL and disability outcomes among individuals with injury.

## Data Availability

Not applicable.

## Appendix

### Search strategies

Embase.com:

(‘quality of life’/exp. OR ‘quality of life assessment’/exp. OR ‘health status indicator’/de OR ‘life satisfaction’/de OR ‘functional status assessment’/de OR ‘Functional Assessment Inventory’/de OR ‘Functional Independence Measure’/de OR ‘Health Assessment Questionnaire’/de OR (‘health status’/de AND ‘rating scale’/de) OR (((quality OR satisf*) NEAR/3 (life OR wellbeing OR well-being)) OR hrql OR hrqol OR ((‘health status’ OR disabilit* OR functional*-independen* OR Functional-Assess* OR Functional-status* OR Functioning OR sickness-impact OR health-utilit*) NEAR/3 (indicator* OR eval* OR assess* OR measure* OR profile* OR index* OR Classification*)) OR ((‘Short Form’ OR SF) NEXT/1 (36 OR 20 OR 12 OR 6)) OR sf36 OR sf20 OR sf12 OR sf6 OR health-profile* OR euroqol OR eq-5d OR hui-2 OR hui2 OR hui-3 OR hui3 OR QWB OR WHODAS-II OR WHODAS-2 OR who-das-ii OR who-das-2):ab,ti) AND (‘injury’/de OR ‘childhood injury’/de OR ‘injury severity’/de OR ‘accidental injury’/de OR ‘injury scale’/de OR ‘multiple trauma’/de OR (injur* OR trauma*):ab,ti) AND (‘cohort analysis’/de OR ‘longitudinal study’/de OR ‘prospective study’/de OR ‘retrospective study’/de OR (cohort* OR longitudinal* OR prospectiv* OR retrospectiv*):ab,ti) NOT ([Conference Abstract]/lim) AND [English]/lim AND [2010–2018].

Medline Ovid:

(Quality of Life/ OR Health Status Indicators/ OR Disability Evaluation/ OR (((quality OR satisf*) ADJ3 (life OR wellbeing OR well-being)) OR hrql OR hrqol OR ((health status OR disabilit* OR functional*-independen* OR Functional-Assess* OR Functional-status* OR Functioning OR sickness-impact OR health-utilit*) ADJ3 (indicator* OR eval* OR assess* OR measure* OR profile* OR index* OR Classification*)) OR ((Short Form OR SF) ADJ (36 OR 20 OR 12 OR 6)) OR sf36 OR sf20 OR sf12 OR sf6 OR health-profile* OR euroqol OR eq-5d OR hui-2 OR hui2 OR hui-3 OR hui3 OR QWB OR WHODAS-II OR WHODAS-2 OR who-das-ii OR who-das-2).ab,ti.) AND (“Wounds and Injuries”/ OR Injury Severity Score/ OR Multiple Trauma/ OR (injur* OR trauma*).ab,ti.) AND (exp Cohort Studies/ OR (cohort* OR longitudinal* OR prospectiv* OR retrospectiv*).ab,ti.) AND english.la.

Limit 2010–2018.

PsycINFO Ovid:

(“Quality of Life”/ OR Disability Evaluation/ OR (((quality OR satisf*) ADJ3 (life OR wellbeing OR well-being)) OR hrql OR hrqol OR ((health status OR disabilit* OR functional*-independen* OR Functional-Assess* OR Functional-status* OR Functioning OR sickness-impact OR health-utilit*) ADJ3 (indicator* OR eval* OR assess* OR measure* OR profile* OR index* OR Classification*)) OR ((Short Form OR SF) ADJ (36 OR 20 OR 12 OR 6)) OR sf36 OR sf20 OR sf12 OR sf6 OR health-profile* OR euroqol OR eq-5d OR hui-2 OR hui2 OR hui-3 OR hui3 OR QWB OR WHODAS-II OR WHODAS-2 OR who-das-ii OR who-das-2).ab,ti.) AND (“Injuries”/ OR (injur* OR trauma*).ab,ti.) AND (Cohort Analysis/ OR Longitudinal Study.md. OR Prospective Study.md. OR Retrospective Study.md. OR (cohort* OR longitudinal* OR prospectiv* OR retrospectiv*).ab,ti.) AND english.la.

Limit 2010–2018.

Web of science:

TS = (((((quality OR satisf*) NEAR/2 (life OR wellbeing OR well-being)) OR hrql OR hrqol OR ((“health status” OR disabilit* OR functional*-independen* OR Functional-Assess* OR Functional-status* OR Functioning OR sickness-impact OR health-utilit*) NEAR/2 (indicator* OR eval* OR assess* OR measure* OR profile* OR index* OR Classification*)) OR ((“Short Form” OR SF) NEAR/1 (36 OR 20 OR 12 OR 6)) OR sf36 OR sf20 OR sf12 OR sf6 OR health-profile* OR euroqol OR eq-5d OR hui-2 OR hui2 OR hui-3 OR hui3 OR QWB OR WHODAS-II OR WHODAS-2 OR who-das-ii OR who-das-2)) AND ((injur* OR trauma*)) AND ((cohort* OR longitudinal* OR prospectiv* OR retrospectiv*))) AND DT = (article) AND LA = (english)

Limit 2010–2018.

## Abbreviations

AIS: Abbreviated Injury Scale
FIM: Functional Independence Measure
GOS: Glasgow Outcome Scale
GOSE: Extended Glasgow Outcome Scale
HRQL: Health-Related Quality of Life
HUI 3: Health Utilities Mark III
ICF: World Health Organization International Classification of Functioning, Disability and Health
ICU: Intensive Care Unit
ISS: Injury Severity Score
MCS: Mental Component Score
NISS: New Injury Severity Score
PCS: Physical Component Score
PedsQL: Paediatric Quality of Life Inventory Generic Core Scales
SF-12: Medical Outcome Study Short Form-12 items
SF-36: Medical Outcome Study Short Form-36 items
WHO: World Health Organization
WHODAS: World Health Organization Disability Assessment Schedule
